# Cross-sectional serosurvey of *Coxiella burnetii* in healthy cattle and sheep from extensive grazing system in central Italy

**DOI:** 10.1017/S0950268819002115

**Published:** 2020-01-20

**Authors:** G. Barlozzari, M. Sala, F. Iacoponi, C. Volpi, N. Polinori, P. Rombolà, F. Vairo, G. Macrì, M. Scarpulla

**Affiliations:** 1 Istituto Zooprofilattico Sperimentale del Lazio e della Toscana “M. Aleandri”, Rome, Italy; 2 Regional Service for Surveillance and Control of Infectious Diseases (SERESMI), National Institute for Infectious Diseases “Lazzaro Spallanzani” IRCCS, Rome, Italy

**Keywords:** Cattle, *Coxiella burnetii*, Italy, Q fever, seroprevalence, sheep

## Abstract

A cross-sectional survey was carried out to estimate the seroprevalence of *Coxiella burnetii* in extensively grazed cattle and sheep from central Italy and to identify the related risk factors. Data on notified human Q fever cases in the area were also collected and described. A two-stage cluster sampling was performed. A total of 5083 animals (2210 cattle; 2873 sheep) belonging to 186 farms (92 herds; 94 flocks) were tested for the presence of antibodies against *C. burnetii* using a commercial enzyme-linked immunosorbent assay kit. The prevalence at the animal-level resulted three times higher in sheep compared to cattle (37.8% *vs.* 12.0%; *χ*^2^ = 270.10, *P* < 0.001). The prevalence at the herd-level was also higher in sheep than in cattle (87.2% *vs.* 68.5%; *χ*^2^ = 9.52, *P* < 0.01). The multivariate analysis showed a higher risk of seropositivity for cattle aged 67–107 months (OR 2.79, 95% CI 1.86–4.18), cattle >107 months of age (OR 2.07, 95% CI 1.36–3.14) and mixed breed cattle (OR 1.74, 95% CI 1.11–2.72). A herd size >92 animals was recognized as herd-level risk factor in cattle (OR 6.88, 95% CI 1.67–28.37). The risk of being seropositive was double in sheep belonging to flocks >600 animals (odds ratio (OR) 2.04, 95% CI 1.63–2.56). Sheep were confirmed to be the most exposed species. Nevertheless, the prevalence observed in cattle also suggests the potential involvement of this species in the circulation of the pathogen in the area. Seven confirmed human Q fever cases were reported. In five out of seven cases there was at least one exposed herd within a 5 km buffer. Even though the source of the infection was not identified, the possibility of *C. burnetii* circulating in the livestock and human population in the study area cannot be overlooked. The integration between veterinary and human surveillance will be crucial to understand the spread of this zoonosis and to support the adoption of appropriate control measures.

## Introduction

*Coxiella burnetii* is an intracellular zoonotic pathogen responsible for Q fever in humans and coxiellosis in domestic and wild mammals. In humans, Q fever is associated with a wide clinical spectrum, from asymptomatic or mildly symptomatic seroconversion to fatal disease. Moreover, in humans acute and chronic Q fever are frequently misdiagnosed and underreported. Farm animals and pets are the main reservoirs of infection and the transmission to humans is mainly accomplished through inhalation of contaminated aerosols [1]. In domestic ruminants, the infection may be asymptomatic or symptomatic. When symptomatic, it may cause epidemic abortion in sheep and goats as well as sporadic abortion, infertility and subclinical mastitis in cattle [2–4]. *C. burnetii* infection can cause significant economic losses in livestock and high health care costs related to diagnosis, long term therapies, hospitalization and working days lost [5, 6]. When abortions occur, high concentrations of *C. burnetii* are found in placenta and birth products of infected animals. Still, the shedding can also take place during normal deliveries [7]. The shedding dynamics differ among ruminants in terms of routes of excretion, load, duration and frequency. In cattle, the bacterium is shed almost exclusively in milk. In goats, it is shed mostly in milk, with a minority shedding it in vaginal mucus or faeces. Sheep result heavily infected and shed the bacterium in faeces, vaginal mucus and milk [7–9]; this could explain why human outbreaks of Q fever are more often related to ovine flocks than to bovine herds. In the 2007–2009 period, large community outbreaks of Q fever occurred in the Netherlands, with over 3500 notified cases in the Dutch population. Proximity to aborting small ruminants and the presence of a large number of susceptible humans were identified as the main causes of the Q fever outbreaks in humans [8]. Thus, the identification of shedders is crucial to avoid infections in humans and to prevent the diffusion among farmed animals. The identification of shedders through direct diagnostic tests is an expensive process that is not yet completely standardized. A fourfold rise in antibody titres could be used to accurately identify acutely infected animals as well, even if this approach is also costly and might not be practical with large sample sizes [9]. Serological tests (indirect immunofluorescence assay – IFA, complement fixation test – CFT and enzyme-linked immunosorbent assay – ELISA) are recommended for seroprevalence studies. CFT has a weak sensitivity compared to other methods and no IFA is commercially available for ruminants to our knowledge, therefore ELISA tests are generally preferred also for practical reasons [10, 11]. Seropositivity to *C. burnetii* is not strongly correlated with the shedding of the bacterium. In fact, some shedders may be seronegative. Due to this, the serology cannot be used to estimate the real contamination rate of the herds, but it is a valuable tool for the screening of the herds and flocks [12–15]. The sensitivity and specificity of milk and blood ELISA are not significantly different, but blood ELISA is necessary for the studies involving non-lactating cattle [13, 16]. Studies have been carried out on ruminants to define the relationship between the specific serological response phase (phase I/II) and the acute/chronic infection stage in analogy with humans. The attempts to classify the shedding pattern based on these studies allowed to classify some groups of animals but no satisfactory results have been achieved at the individual level so far [17–19]. Serosurveys were performed to evaluate the exposure to *C. burnetii* among healthy ruminants in Europe, US, Africa and Asia. These serosurveys reported prevalence at the animal-level ranging from 11% to 19.5% in sheep and 6.2% to 14.4% in cattle. The reported prevalence at the herd-level ranged from 38.7% to 74% in sheep, and from 16.7% to 71% in cattle [20–28]. In Italy, the data on Q fever prevalence in ruminants are mainly related to animals with reproductive disorders and particularly to those with abortion as the major clinical problem [29–32]. The Commission Implementing Regulation (EU) 2018/1882 included Q fever among the diseases enlisted within the ‘e’ category, which need surveillance and specific rules for notification and reporting as defined by the Regulation (EU) 2016/429 (‘Animal Health Law’). Nevertheless, the surveillance and reporting of *C. burnetii* in animals are not harmonized in EU. In Italy, the outbreak management is currently regulated by the Presidential Decree 320/1954. The Decree states that specific restrictive measures on farmed animals and milk products must be adopted only when human Q fever cases are related to the exposure to infected animals. In EU, Q fever is a mandatory notifiable disease in humans and all cases are reported through The European Surveillance System. The definition of ‘Q fever case’ is established precisely within the EU regulation, but the surveillance framework is not harmonized across Member States [33, 34]. In Italy, Q fever is a notifiable disease regulated under the Informative System for Infectious Diseases (DM 15/12/90). Q fever cases must be notified to the Local Public Health Authority and reported annually to the Regional Public Health Authority and to the Ministry of Health. Until 2015, Q fever cases were reported within the generic group of ‘rickettsial infections’. Since 2015, the overall surveillance system has been strengthened in the Lazio region to cope with the 2015–2016 Extraordinary Jubilee. Every single Q fever case was immediately reported to the Regional Health Authority by the Local Public Health authority. Few information on *C. burnetii* diffusion in domestic ruminants is available in the Lazio region, as the only existing data concern the differential diagnosis performed in the case of abortion. Furthermore, these data are likely underestimated due to the low notification rate of abortions by farmers. Recently, some polymerase chain reaction (PCR)-positive samples from aborted animals bred in the Lazio region were typed by Multispacer Sequence Typing (MST). The resulting MST genotypes (ST32 and ST12) were identical to those previously detected in clinical human samples from other countries [35]. This evidence emphasizes the need for reliable information on *C. burnetii* diffusion in this area. Consolidated prevalence data are useful to quantify the exposure of the animal population to pathogens and represent the first step within the decision process of the health authorities. For this purpose, a cross-sectional study was carried out in extensively grazing cattle and sheep in the Lazio region, where this farming system is commonly practiced. The prevalence at animal- and herd-level of *C. burnetii* was estimated and the related risk factors were identified. Data on notified human Q fever cases in the area were also collected and described.

## Methods

### Study area and sampling

This serosurvey was carried out in the 2013–2014 period in the province of Rome (Metropolitan City). The province covers almost one-third of the territory of the Lazio region, central Italy, including the flat area of the Roman country, the Tiber Valley and several hilly and mountainous areas. With 5.4 km^2^ and 4.4 million inhabitants, the Metropolitan City includes the city of Rome, which is the most populated municipality in Italy and the fourth most populous city in the European Union. Rome is also the greenest city in Europe, with 63.8% of its territory covered by green areas. A portion of these areas is agricultural and pastureland, extending from centre to the borders of the Metropolitan City, where cattle and sheep are reared in an extensive grazing system. The beef cattle breeding scheme adopted in the area includes both purebred and mixed breed animals. Purebred animals represent the high-value breeding stock held under controlled conditions on permanent pastures; mixed breed animals which are bred for the commercial production of beef calves, are held under free grazing conditions on wider pastures. Cattle and sheep blood samples were collected by government veterinarians within the national eradication programme of brucellosis. Sera were separated by centrifugation for 10 min at 1000 × *g* and stored at −20 °C until examination at the Regional State Laboratory where the authors operate.

### Serological tests

Cattle and sheep sera were analysed to detect anti-phase I and anti-phase II antibodies against *C. burnetii* by an ELISA prepared with *C. burnetii* strain isolated from ruminants (ID Screen Q Fever Indirect Multi-speciesIdvet, Grabels, France). In the internal validation sheet, the manufacturer declared a sensitivity of 100% (CI 95%: 88.65%–100%) and a specificity of 100% (CI 95%: 98.49%–100%). The results were expressed in an optical density sample/positive control (S/P) ratio, measured at 450 nm. The samples presenting an S/P percentage >50% were considered as positive; those with 40% ⩽ S/P% < 50% were considered as doubtful; otherwise samples were considered as negative. In this study, doubtful results were considered as negative.

### Study population and study design

Data on the animal population were obtained from the National Animal Registry Database (Banca Dati Nazionale: BDN) defining a study population of 206 000 sheep belonging to 461 farms and 30 000 cattle from 1627 farms. Only extensive grazing animals were considered as target population for both species. The study population was considered as homogeneous for infection risk as they were all herds from extensive farming under similar managing conditions. A two-stage cluster sampling was performed, with herds as primary units and animals as secondary units, to estimate the prevalence of animals with a detectable level of antibodies against *C. burnetii*. The following assumptions were used for both species: 30% expected prevalence, 95% confidence level, ±5% or standard error and 0.2 intra-cluster correlation coefficient (low within-cluster homogeneity). The calculated minimum sample size for each species was at least 2194 animals from 73 clusters (herds). An *a priori* number of 30 animals was set to be tested in each herd. The herds and animals to be tested were selected from the list of samples stored during the 2013 and 2014 national brucellosis eradication programme, using a simple random sampling (SRS) method. As the herds were selected by SRS sampling a fixed number of animals which was not proportional to the herd size, the prevalence estimates (*P*_adj_) and 95% CI were adjusted using weights to account for the cluster sampling design employed using the following formula:
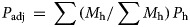


where *P*_h_ stands for the proportion of positive individuals in each selected herd (No. positive/No. tested), *M*_h_ stands for the number of animals present in each selected herd and ∑*M*_h_ stands for the total number of animals present in all the tested herds [36].

The sampled animals were clinically healthy cattle and sheep, aged at least one year, with no history of previous vaccination against coxiellosis. Information on age, sex, breed, herd size and other species housed in the holdings were extrapolated from BDN for each randomly sampled cattle serum. The total number of farmed ruminants and the other animal species housed in the holdings were the only information available for sheep in BDN.

### Statistical analysis

The prevalence at animal-level was separately calculated in cattle and sheep, considering the herd size as the weighting factor. The prevalence at herd-level was calculated for each species as the proportion of herds with at least one positive ELISA test on the total number of tested herds; 95% CI for prevalence were estimated by binomial distribution. The Chi square (*χ*^2^) test was used to compare prevalence estimates between cattle and sheep. The following animal-level risk factors were considered: herd size (No. of cattle/sheep in the herd), species housed in the holding (cattle, sheep or both), age (in months), sex (male, female) and breed (mixed breed – MB, Maremmana – MRN, Charolaise – CHL, other breeds – other). The effect of the recorded risk factors on the outcome was assessed at animal- and herd-levels. Age and herd size were reported as medians and interquartile range (IQR, 75°–25° centile) after the Kolmogorov–Smirnov test for normality distribution. These two variables were divided into classes following the quantile classification method. To evaluate linearity, categories were created and the Log odds ratios (ORs) obtained from the univariate analysis performed were plotted against the midpoint of the category [37]. Discrete and qualitative data were reported as frequencies and percentages (%) respectively. The effect of the risk factors on the individual presence of detectable antibodies against *C. burnetii* was analysed using univariate logistic regression models. After excluding the associated variables (*χ*^2^ test), the risk factors with a bivariate *P* value ⩽0.25 were included in a multivariate backward logistic model [38]. The likelihood ratio *χ*^2^ test was applied in order to select the best fitting model. The herd size and other animal species housed in the holdings were considered as risk factors for the herd-level analysis. The reference classes (*ref*) for risk factors were set as a herd size <30, age <37 months, male sex and MRN breed for cattle. The reference classes adopted for sheep were: herd size <176 and only-sheep housing. The strength of the association between the risk factors and the outcome was estimated using the ORs with 95% CI at animal- and herd-levels. A *P* value <0.05 was considered statistically significant. All statistical analyses were performed by Stata/SE version 12 for Windows (StataCorp LP, TX, USA).

### Humans

Q fever cases in humans were notified by the doctors to the Local Public Health Authority. The Local Public Health Authority annually reported the notifications to the Regional Public Health Authority. Following the intensified notification flow after the 2015–16 Jubilee, every single case was reported also to Regional Public Health Authority. In accordance with the EU regulation, the cases were classified as: possible, probable or confirmed cases [33]. Demographic data, diagnostic criteria, date of symptom onset and risk factors were also collected and described for each case.

### Mapping

ArcGIS 10.3^®^ was used for mapping. The distribution of the holdings, weighted by the herd size, was used to build a kernel density map using the following parameters: 5 km radius (bandwidth), 1 km^2^ cell size and natural breaks classification (Jenks). The other layers used were: human cases, tested herds and not tested herds within 5 km buffers around the human cases.

## Results

### Cattle

A total of 2210 animals were tested for *C. burnetii*. The median age of the tested animals was 5.47 years (IQR 5.94), and 93.03% were female. The most frequent breeds were MB (55.7%) and MRN (15.57%). The prevalence at animal-level was 12.0% (95% CI 9.28–14.77) (Table 1). At the univariate analysis, the following animal-level risk factors were identified: age ≥67 months (OR 2.72, 95% CI 1.82–4.07), female sex (OR 2.28, 95% CI 1.11–4.71) and MB (OR 1.68, 95% CI 1.08–2.61). MRN breed was recognized as a protective factor. The presence of antibodies against *C. burnetii* in the animals did not result statistically associated neither with the herd size, nor with the presence of other farmed species in the holdings. In the multivariate analysis, only age and breed were maintained as significant risk factors within the best fitting model, since the effect of sex was lost in the first step of the backward deletion procedure. The animal-level risk factors confirmed in the best fitting model were: age classes 67–107 months (OR 2.79, 95% CI 1.86–4.18), >107 months (OR 2.07, 95% CI 1.36–3.14) and MB (OR 1.74, 95% CI 1.11–2.72) (Table 2).

**Table 1.** Animal-level and herd-level prevalence of *C. burnetii* in cattle and sheep

	Animals/herds (*n*)	Positive (*n*)	Negative (*n*)	Prevalence % (95% CI)	Weighted prevalence % (95% CI)
Animals					
Cattle	2210	238	1972	10.77 (9.51–12.14)	12.02 (9.28–14.77)
Sheep	2873	859	2014	29.9 (28.23–31.61)	37.82 (33.32–42.32)
Herds					
Cattle	92	63	29	68.48 (57.9–77.7)	
Sheep	94	82	12	87.23 (78.8–93.2)	

CI, confidence interval.

**Table 2.** Univariate and multivariate analyses of the animal-level prevalence of *C. burnetii* in cattle

Risk factors	Prevalence % (*n* seropositive/total)	Univariate analysis OR (95% CI)	Best fitting multivariate model OR (95% CI)
Age (months)			
<37	6.53 (38/582)	*ref*	*ref*
37–66	8.82 (47/533)	1.38 (0.89–2.16)	1.42 (0.91–2.22)
67–107	15.98 (85/532)	2.72 (1.82–4.07)**	2.79 (1.86–4.18)**
>107	12.38 (67/541)	2.02 (1.33–3.07)**	2.07 (1.36–3.14)**
Sex			
M	5.19 (8/154)	*ref*	*–*
F	11.18 (230/2057)	2.28 (1.11–4.71)*	
Breed			
MRN	7.27 (25/344)	*ref*	*ref*
MB	11.62 (143/1232)	1.68 (1.08–2.61)*	1.74 (1.11–2.72)*
CHL	10.67 (19/178)	1.52 (0.82–2.85)	1.61 (0.86–3.03)
Other	11.49 (50/435)	1.66 (1.00–2.74)	1.76 (1.06–2.92)*
Herd size			
<30	9.39 (49/522)	*ref*	–
30–46	11.52 (104/903)	1.26 (0.88–1.80)	
47–92	9.59 (23/240)	1.02 (0.61–1.72)	
>92	11.38 (62/545)	0.24 (0.83–1.84)	
Animal species			
Cattle	10.58 (187/1768)	*ref*	–
Cattle + sheep	2.88 (51/442)	1.10 (0.79–1.53)	
Total cattle (*n*)	2210		

OR, odds ratio; CI, confidence interval; *ref*, reference category.

*P < 0.05; **P < 0.01.

A total of 92 herds were tested. The median herd size was 41.5 animals (IQR 57.75) and the median number of ruminants housed was equal to 49 (IQR 102.5). Seventy-three herds (79.4%) housed only cattle and 19 (20.6%) both cattle and sheep. Overall, 6511 heads were housed within the selected herds, accounting for 21.7% of the target population living in the study area. The prevalence at herd-level was 68.5% (95% CI 57.9–77.7) (Table 1). An increasing risk for herds to result positive for antibodies against *C. burnetii* was observed in relation to the herd size. Notably at the univariate analysis, herd size >92 showed about seven times higher risk (OR 6.88, 95% CI 1.67–28.37) compared to the reference class. No association was found between the presence of at least one animal with detectable antibodies against *C. burnetii* and the presence of other farmed animal species in the same holding (Table 3).

**Table 3.** Univariate analysis of of the herd-level prevalence of prevalence of *C. burnetii* in cattle

Risk factors	Prevalence % (*n* seropositive/total)	Univariate analysis OR (CI 95%)
Herd size		
<30	45.16 (14/31)	*ref*
30–46	76.19 (16/21)	3.89 (1.14–13.27)*
47–92	80 (16/20)	4.86 (1.32–17.89)*
>92	85 (17/20)	6.88 (1.67–28.37)**
Animal species		
Only cattle	68.49 (50/73)	*ref*
Cattle + sheep	68.42 (13/19)	0.99 (0.34–2.95)
Total herds (*n*)	92	

OR, odds ratio; CI, confidence interval; *ref*, reference category.

*P < 0.05; **P < 0.01.

### Sheep

A total of 2873 animals were tested for *C. burnetii*. The prevalence at animal-level was 37.8% (95% CI 33.3–42.3) (Table 1). At the univariate analysis, the risk of being seropositive was double in sheep belonging to flocks >600 animals (odds ratio (OR) 2.04, 95% CI 1.63–2.56) compared to the reference class (flock size <176). No statistical association was observed between the presence of detectable antibodies against *C. burnetii* in the animals and the presence of other farmed animal species in the holdings.

A total of 94 flocks were tested. The median flock size was 360 animals (IQR 392.5) and the median number of ruminants was 365.5 (IQR 446.75). Sixty-four flocks (68.1%) housed only sheep and 30 (31.9%) housed both sheep and cattle. Overall, 42 596 sheep were farmed within the tested flocks, accounting for 20.7% of the target population living in the study area. The prevalence at herd-level was 87.2% (95% CI 78.8–93.2) (Table 1). The univariate analysis showed that the probability that a flock was positive for antibodies against *C. burnetii* did not depend neither from the flock size, nor from the presence of other animal species housed in the holding.

### Prevalence in cattle and sheep

The weighted prevalence at animal-level was three times higher in sheep compared to cattle (37.8% *vs.* 12.0%; *χ*^2^ = 270.10, *P* < 0.001); the prevalence at herd-level resulted significantly higher in sheep than in cattle (87.2% *vs.* 68.5%; *χ*^2^ = 9.52, *P* < 0.01).

### Humans

**Fig. 1.**
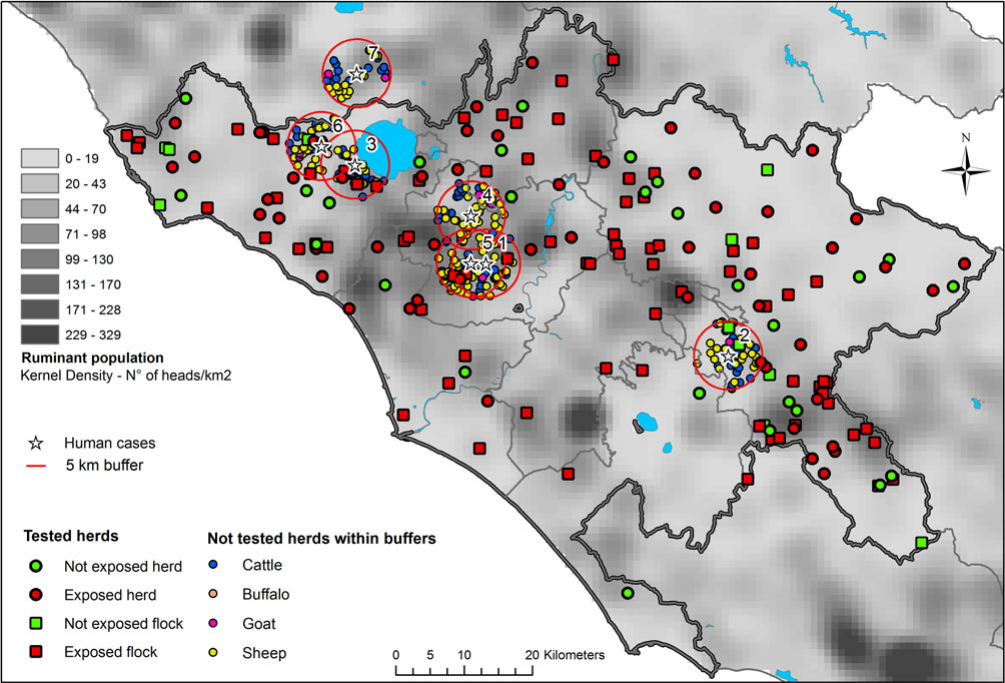
Human Q fever cases and tested herds in the study area; not tested herds within 5 km buffers around the human cases.

Seven confirmed human Q fever cases were reported. Six cases were localized in the rural area surrounding the city of Rome and one in the province of Viterbo, close to the border of the province of Rome (Fig. 1). The date of onset of clinical disease was from November 2016 to November 2017. All cases had positive serology following clinical manifestations. Five cases were male and one female. The proximity to small ruminant holdings was explicitly referred to as risk factor in three cases. None of the cases was reported to work in the sampled farms. In five out of seven cases there was at least one exposed herd within a 5 km buffer. One case was living 6.8 km far from exposed herds. The case falling within the province of Viterbo was living 0.327 km far from the nearest herd. All cases fell within cells having a density of ruminants from 9.8 to 156.9 heads per km^2^. The distance to the nearest herd is comprised from 0.169 to 1.024 km and in two cases the nearest herd was an exposed herd (Table 4). The infection source was not identified.

**Table 4.** Human Q fever cases in the study area

Case Id	Reported risk factors	E flocks in a 5 km buffer (*n*)	E herds in a 5 km buffer (*n*)	Density of ruminants (*n* heads/km^2^)	Distance from the nearest herd (km)/herd type
1	Goats	3	1	156.9	0.293/sheep
2	Sheep, wild animals	0	1	9.8	1.024/sheep
3	–	3	2	62.0	0.324/cattle^a^
4	Dogs	1^b^	1^b^	67.8	0.169/goat
5	Sheep	1	2	149.3	0.525/cattle
6	Dogs	1	2	37.2	0.478/cattle^a^
7	–	–	–	118.9	0.327/sheep

E, exposed.

a The nearest herd is an exposed herd. ^b^6.8 km far.

## Discussion

In this study, the prevalence at animal-level of *C. burnetii* was 12.0% in healthy cattle. Other studies carried out in Italy reported prevalence ranging from 14.4% to 22% in healthy cows, whereas a 44.9% prevalence was reported in cows with abortion [20, 30]. We found that the risk of testing positive for antibodies against *C. burnetii* increased in mixed breed cattle compared to the reference class (MRN breed). This result is consistent with the different exposure to this pathogen that apparently occurred, by considering the beef cattle breeding scheme adopted in the study area. MRN breed animals are purebred subjects, belonging to the high-value breeding stock used for the production of commercial female breeders. The purebred animals are kept under more controlled conditions consisting of fenced permanent grazing pastures, ensuring levels of higher biosecurity. Conversely, the mixed breed animals belonging to the category of breeders, used for the commercial production of beef calves, are held in conditions of free grazing on wider pastures where contacts with animals from different herds often occur. For this reason they could be probably more at risk for the transmission of the infection. In a previous study, the Friesian breed resulted a risk factor for the presence of antibodies against *C. burnetii* [23]. In the present study, the probability of having detectable antibodies against *C. burnetii* increased with the age (>67 months). This result is in line with other studies indicating that the odds of being infected are higher in older animals [23, 39]. This finding could be explained with a higher probability of being exposed to the pathogen over the years. In a study carried out in Italy, the prevalence at animal-level of *C. burnetii* was found to be influenced by herd management. In detail, it was 19.6% in those herds housed in winter and unhoused in spring; 13.2% in permanently housed animals and 1.9% in unhoused cattle [20]. In Spain, Ruiz-Fons *et al*. reported a 6.7% prevalence at animal-level in a semi-extensive grazing system [22]. By contrast, in this study the prevalence at animal-level was higher than those observed by the above-mentioned authors. The higher prevalence of *C. burnetii* observed in the area could be due to different breeding methods, different densities of receptive animal species, and to the eco-environmentally favourable conditions for the exposure to the pathogen. Due to the lack of information currently available for these potential risk factors, further *ad hoc* investigations should be performed to compare the prevalence of *C. burnetii* among different herd management conditions in the study area. In this study, the prevalence at herd-level was 68.5% in cattle. In Italy, 4.4% herd-level prevalence was estimated using CFT [40]. In Spain 43.0% herd-level prevalence was reported in cattle [22]. The wide range of the seroprevalence reported by those studies could be due to the distinct epidemiological scenarios. Nevertheless, the prevalence reported was also affected by the performances of the assays employed. With regards to the ELISA employed in this study, the manufacturer reported a 100% sensitivity (CI 95%: 88.65%–100%) and a 100% specificity (CI 95%: 98.49%–100%). In the worst-case scenario, considering the lower confidence limit of the sensitivity (88.65%) provided by the manufacturer, the prevalence estimates provided in our study were probably underestimated compared to the true prevalence. By converse, the lower confidence limit of the specificity (98.49%) suggested a low probability of occurrence of false negative results, in accordance with what is well known for a highly specific test such as the CFT. Thus, an overestimation of prevalence was unlikely to have occurred. Anyway, by considering the lower confidence limits of both specificity and sensitivity of the ELISA test employed, the positive predictive value obtained was expected to be very high, considering also the high seroprevalence observed. CFT is not so suitable for serological studies since it fails to detect cases when anti-complementary substances are present in sera. Moreover, in ruminants some antibodies are not revealed by CFT because only IgG1 antibodies are known to fix the complement. Furthermore, CFT titers may be reduced due to the presence of IgG2 and IgM antibodies which can suppress complement fixation by IgG1 antibodies [10, 41]. In our study, the risk of having at least one animal with detectable antibodies against *C. burnetii* was seven times higher in herds with more than 92 heads. This result is consistent with the hypothesis that animals from larger herds were more likely to come into contact with infected individuals. In particular, an increasing animal density could result in a higher probability of being directly exposed to *C. burnetii* during the deliveries. Antibodies against *C. burnetii* were detected in sheep from an extensive farming system with 37.8% prevalence at animal-level. Recent surveys carried out on healthy sheep in northwest Italy and in Sicily reported 16.3% and 18.0% individual prevalence respectively [27, 28]. Nine percent prevalence at animal-level was estimated using a Nine Mile ELISA test in sheep with abortion [29]. In this study, the risk of being exposed to *C. burnetii* resulted double for sheep belonging to flocks with >600 animals. The higher risk of being seropositive for animals from larger flocks may be related to the greater number of lambing females which increases the total population at risk and, subsequently, the risk of introduction and transmission of this pathogen. In our study, the prevalence at herd-level was 87.2% in sheep. The probability of having at least one positive animal did not depend neither from the flock size, nor from the presence of other animal species housed in the holding. This finding is not in agreement with those studies reporting the flock size as herd-level risk factor for *C. burnetii* [27]. The prevalence at animal-level of *C. burnetii* resulted three times higher in sheep compared to cattle (37.8% *vs.* 12.0%); the prevalence at herd-level was higher in sheep than in cattle (87.2% *vs.* 68.5%) as well. These findings could be explained with the higher infection load in sheep and with the several ways of shedding that occurs in this species, resulting in a substantial exposure to the pathogen [7, 14, 42]. Furthermore, the gregarious aptitude of sheep enhances the contacts among animals and so the probability of being exposed to *C. burnetii* through direct and indirect routes also in an extensive grazing system. Our results highlight the considerable diffusion of *C. burnetii* in the study area. It was unexpected to find such a high prevalence in an extensive grazing system. This evidence led us also to hypothesize a possible involvement of ticks in the epidemiology of the pathogen in the area. Notoriously, ticks represent a secondary route of infection of *C. burnetii* in ruminants. The vector competence has been demonstrated for many hard and soft tick species even if the vector capacity could be low in field conditions. Conversely, the tick-borne transmission of the bacterium seems to be more efficient in wild animals. In addition, the recent discovery of *Coxiella*-like bacteria, endosymbionts of several tick species, imply the possibility that they could be transmitted to vertebrates during the blood meal and misidentified as *C. burnetii* when vertebrates are screened using either direct or indirect tests [43, 44]. Moreover, pastures and vegetation can be frequently shared by domestic and wild animals in an extensive grazing system, thus facilitating the transmission of a huge variety of pathogens from the wild reservoirs, through the direct or indirect route [45–47]. Sheep were confirmed to be the most exposed species at both animal- and herd-levels. However, the seroprevalence observed in cattle suggests also a potential involvement of this species in the circulation of the bacterium in the study area. The presence of domestic ruminants substantially exposed to an air-borne zoonotic pathogen indicates that also humans can be potentially exposed and become infected. In the study area, five out of seven confirmed human Q fever cases had at least one exposed herd within a 5 km buffer. The distance to the nearest herd is comprised from 0.169 to 1.024 km and in two cases the nearest herd is an exposed herd. In a recent review, the highest risk of infection for humans occurred within 5 km of infected farms in rural areas [48]. Even if the infection source was not identified in any of those cases, the possibility of *C. burnetii* circulating in the livestock and human population in the study area cannot be overlooked. These findings suggest the need to implement surveillance and control systems based on a ‘One Health’ approach, involving the systematic notification of cases in both humans and animals. The integration between veterinary and human surveillance will be crucial to understand the spread of this zoonosis and to support the adoption of appropriate control measures. Collaborative surveillance platforms where cases and outbreaks are reported could facilitate the epidemiological investigations and define the infection risk. Efforts were made in the Lazio region to develop such a platform following the collaboration between the IZSLT and the Seresmi. However, further studies are needed in the study area to collect and characterize the strains circulating in ruminants and to compare them with those isolated from humans, through a Multiple-Locus Variable number tandem repeat Analysis/MST approach or Next-Generation Sequencing. Such an approach would be useful to a better understanding of the epidemiology of *C. burnetii* and to trace the strains in the case of outbreaks.

## References

[1] Angelakis E and Raoult D (2010) Q Fever. Veterinary microbiology 140, 297–309. 19875249 10.1016/j.vetmic.2009.07.016

[2] Barlow J (2008) *Coxiella burnetii* associated reproductive disorders in domestic animals – a critical review. Acta Veterinaria Scandinavica 39, 1–9. 10.1186/1751-0147-55-13 PMC3577508 23419216

[3] Agerholm JS (2013) *Coxiella burnetii* associated reproductive disorders in domestic animals – a critical review. Acta Veterinaria Scandinavica 55, 13. 23419216 10.1186/1751-0147-55-13 PMC3577508

[4] De Biase D (2018) *Coxiella burnetii* in infertile dairy cattle with chronic endometritis. Veterinary Pathology 55, 539–542. 29566608 10.1177/0300985818760376

[5] Garner MG (1997) A review of Q fever in Australia 1991–1994. Australian and New Zealand Journal of Public Health Wiley-Blackwell 21, 722–730. 10.1111/j.1467-842x.1997.tb01787.x 9489189

[6] Van Asseldonk MAPM, Prins J and Bergevoet RHM (2013) Economic assessment of Q fever in the Netherlands. Preventive Veterinary Medicine 112, 27–34. 23866818 10.1016/j.prevetmed.2013.06.002

[7] Rodolakis A (2007) Comparison of *Coxiella burnetii* shedding in milk of dairy bovine, caprine, and ovine herds. Journal of Dairy Science 90, 5352–5360. 18024725 10.3168/jds.2006-815

[8] Roest HIJ (2011) The Q fever epidemic in the Netherlands: history, onset, response and reflection. Epidemiology and Infection 139, 1–12. 20920383 10.1017/S0950268810002268

[9] Muleme M (2017) Peripartum dynamics of *Coxiella burnetii* infections in intensively managed dairy goats associated with a Q fever outbreak in Australia. Preventive Veterinary Medicine 139, 58–66. 28364833 10.1016/j.prevetmed.2017.02.006

[10] Sidi-Boumedine K (2010) Development of harmonised schemes for the monitoring and reporting of Q-fever in animals in the European Union. EFSA Scientific Report on Question No EFSA-Q-2009-00511, pp. 48. Published online: May 2010. Available online: http://www.efsa.europa.eu/en/supporting/pub/en-48

[11] Natale A (2012) Comparative immunology, microbiology and infectious diseases old and new diagnostic approaches for Q fever diagnosis: correlation among serological (CFT, ELISA) and molecular analyses. ‘Comparative Immunology, Microbiology and Infectious Diseases’ 35, 375–379. 10.1016/j.cimid.2012.03.002 22463984

[12] Berri M (2001) Relationships between the shedding of *Coxiella burnetii*, clinical signs and serological responses of 34 sheep. Veterinary Record 148, 502–505. 11345992 10.1136/vr.148.16.502

[13] Guatteo R (2007) *Coxiella burnetii* shedding by dairy cows. Veterinary Research 38, 849–860. 17903418 10.1051/vetres:2007038

[14] Rousset E (2009) *Coxiella burnetii* shedding routes and antibody response after outbreaks of Q fever-induced abortion in dairy goat herds. Applied and Environmental Microbiology 75, 428–433. 19011054 10.1128/AEM.00690-08 PMC2620711

[15] EFSA Panel on Animal Health and Welfare and EFSA Panel on Biological Hazards (2010) Scientific Opinion on Q Fever. EFSA Journal 8, 1595.

[16] Paul S (2013) Bayesian estimation of sensitivity and specificity of *Coxiella burnetii* antibody ELISA tests in bovine blood and milk. Preventive Veterinary Medicine 109, 258–263. 23182027 10.1016/j.prevetmed.2012.10.007

[17] Böttcher J (2011) Insights into the dynamics of endemic *Coxiella burnetii* infection in cattle by application of phase-specific ELISAs in an infected dairy herd. Veterinary Microbiology 151, 291–300. 21482042 10.1016/j.vetmic.2011.03.007

[18] Sting R (2013) Quantitative real-time PCR and phase specific serology are mutually supportive in Q fever diagnostics in goats. Veterinary Microbiology 167, 600–608. 24095624 10.1016/j.vetmic.2013.09.015

[19] Lucchese L (2015) IFAT and ELISA phase I/phase II as tools for the identification of Q fever chronic milk shedders in cattle. Veterinary Microbiology 179, 102–108. 25769644 10.1016/j.vetmic.2015.02.010

[20] Capuano F, Landolfi MC and Monetti DM (2001) Influence of three types of farm management on the seroprevalence of Q fever as assessed by an indirect immunofluorescence assay. Veterinary Record 149, 669–671. 11765323 10.1136/vr.149.22.669

[21] Khalili M and Sakhaee E (2009) An update on a serologic survey of Q fever in domestic animals in Iran. American Journal of Tropical Medicine and Hygiene 80, 1031–1032. 19478271

[22] Ruiz-Fons F (2010) Seroepidemiological study of Q fever in domestic ruminants in semi-extensive grazing systems. BMC Veterinary Research 6, 1–6. 20089188 10.1186/1746-6148-6-3 PMC2831013

[23] Mccaughey C (2010) *Coxiella burnetii* (Q fever) seroprevalence in cattle. Epidemiology and Infection 138, 21–27. 19480726 10.1017/S0950268809002854

[24] Scolamacchia F (2010) Serological patterns of brucellosis, leptospirosis and Q fever in Bos indicus cattle in Cameroon. PLoS ONE 5, e8623. 10.1371/journal.pone.0008623 PMC2809085 20098670

[25] Guatteo R (2011) Prevalence of *Coxiella burnetii* infection in domestic ruminants: a critical review. Veterinary Microbiology 149, 1–16. 21115308 10.1016/j.vetmic.2010.10.007

[26] Meadows S (2015) *Coxiella burnetii* seropositivity and associated risk factors in sheep in Ontario, Canada. Preventive Veterinary Medicine 122, 129–134. 26376066 10.1016/j.prevetmed.2015.07.007

[27] Rizzo F (2016) Q fever seroprevalence and risk factors in sheep and goats in northwest Italy. Preventive Veterinary Medicine 130, 10–17. 27435642 10.1016/j.prevetmed.2016.05.014

[28] Villari S (2018) Seroprevalence of *Coxiella burnetii* infection (Q fever) in sheep farms located in Sicily (Southern Italy) and related risk factors. Small Ruminant Research 164, 82–86.

[29] Masala G (2004) Occurrence, distribution, and role in abortion of *Coxiella burnetii* in sheep and goats in Sardinia, Italy. Veterinary Microbiology 99, 301–305. 15066733 10.1016/j.vetmic.2004.01.006

[30] Cabassi CS (2006) Association between *Coxiella burnetii* seropositivity and abortion in dairy cattle of Northern Italy. New Microbiologica 29, 211–214. 17058789

[31] Parisi A (2006) Diagnosis of *Coxiella burnetii*-related abortion in Italian domestic ruminants using single-tube nested PCR. Veterinary Microbiology 118, 101–106. 16891064 10.1016/j.vetmic.2006.06.023

[32] Natale A (2009) First report of bovine Q-fever in north-eastern Italy: preliminary results. Clinical Microbiology and Infection 15, 144–145. 10.1111/j.1469-0691.2008.02154.x 19438637

[33] European Commission, Directorate-General for Health and Food Safety (2018) Commission Implementing Decision (EU) 2018/945 of 22 June 2018 on the communicable diseases and related special health issues to be covered by epidemiological surveillance as well as relevant case definitions, The Official Journal of the European Union L series 170, 1–74.

[34] European Food Safety Authority and European Centre for Disease Prevention and Control (2018) The European Union summary report on trends and sources of zoonoses, zoonotic agents and foodborne outbreaks in 2017. EFSA Journal 16, e05500. doi: 10.2903/j.efsa.2018.5500. PMC7009540 32625785

[35] Di Domenico M (2018) Genetic diversity of *Coxiella burnetii* in domestic ruminants in central Italy. BMC Veterinary Research 14, 171, e05500. doi: 10.2903/j.efsa.2018.5500. PMC5975477 29843709

[36] Ferrari G (2016) Foot and mouth disease vaccination and post-vaccination monitoring: Guidelines. Rome: Food and Agriculture Organization of the United Nations & World Organisation for Animal Health Inc, pp. 82.

[37] Dohoo I, Martin W and Stryhn H (2009) Veterinary Epidemiologic Research, 2nd Edn. Charlottetown, Prince Edward Island, Canada: VER Inc, pp. 865.

[38] Hosmer DW and Lemeshow S (2000) Applied Logistic Regression, 2nd Edn New York: John Wiley & Sons Inc, pp. 373.

[39] Alvarez J (2012) Epidemiological factors associated with the exposure of cattle to *Coxiella burnetii* in the Madrid region of Spain. The Veterinary Journal 194, 102–107. 22534189 10.1016/j.tvjl.2012.02.022

[40] Martini M, Baldelli R and Paulucci De Calboli L (1994) An epidemiological study on Q fever in the Emilia-Romagna Region, Italy. Zentralblatt für Bakteriologie 280, 416–422. 8167437 10.1016/s0934-8840(11)80606-7

[41] Rousset E (2007) Comparative diagnostic potential of three serological tests for abortive Q fever in goat herds. 124, 286–297. 10.1016/j.vetmic.2007.04.033 17532581

[42] Guatteo R (2006) Shedding routes of *Coxiella burnetii* in dairy cows: implications for detection and control. Veterinary Research 37, 827–833. 16973121 10.1051/vetres:2006038

[43] Duron O (2015) The IS1111 insertion sequence used for detection of Coxiella burnetii is widespread in Coxiella -like endosymbionts of ticks. FEMS Microbiology Letters. 362, fnv132. doi: 10.1093/femsle/fnv132. 26269380

[44] Angelakis E (2016) Candidatus coxiella massiliensis infection. Emerging Infectious Diseases 22, 285–288. 26811945 10.3201/eid2202.150106 PMC4734529

[45] Cantas H (2011) Q fever abortions in ruminants and associated on-farm risk factors in northern Cyprus. BMC Veterinary Research 7, 13. 21414196 10.1186/1746-6148-7-13 PMC3070639

[46] Billinis C (2011) Molecular investigation of the occurrence of *Coxiella burnetii* in wildlife and ticks in an endemic area. Small Ruminant Research 147, 190–194. 10.1016/j.vetmic.2010.05.046 20580169

[47] Barlozzari G (2015) First report of Brucella suis biovar 2 in a semi free-range pig farm, Italy. Veterinaria Italiana 51, 151–154. 26129667 10.12834/VetIt.50.3384.1

[48] Clark NJ and Soares Magalhães RJ (2018) Airborne geographical dispersal of Q fever from livestock holdings to human communities: a systematic review and critical appraisal of evidence. BMC Infectious Diseases 18, 218. doi: 10.1186/s12879-018-3135-4. PMC5952368 29764368

